# Outcome assessment of a complex mental health intervention in the workplace. Results from the MENTUPP pilot study

**DOI:** 10.1007/s00420-023-01996-3

**Published:** 2023-07-14

**Authors:** Fotini Tsantila, Evelien Coppens, Hans De Witte, Ella Arensman, Benedikt Amann, Arlinda Cerga-Pashoja, Paul Corcoran, Johanna Creswell-Smith, Grace Cully, Monika Ditta Toth, Birgit Greiner, Eve Griffin, Ulrich Hegerl, Carolyn Holland, Caleb Leduc, Mallorie Leduc, Doireann Ni Dhalaigh, Cliodhna O’Brien, Charlotte Paterson, György Purebl, Hanna Reich, Victoria Ross, Reiner Rugulies, Sarita Sanches, Katherine Thompson, Chantal Van Audenhove, MENTUPP consortium members, Kahar Abula, Birgit Aust, Laura Cox, Luigia D’Alessandro, Grace Davey, Lars De Winter, Kim Dooyoung, Asmae Doukani, Arilda Dushaj, Naim Fanaj, Stefan Hackel, Bridget Hogg, Sharna Mathieu, Margaret Maxwell, Ana Moreno- Alcazar, Karen Mulcahy, Doireann Ni Dhalaigh, Ainslie O’ Connor, Wendy Orchard, Gentiana Qirjako, Saara Rapeli, Sarita Sanches, Andras Szekely, Jaap Van Weeghel, Kristian Wahlbeck, Eva Zsak

**Affiliations:** 1 Centre for Care Research and Consultancy, LUCAS, KU Leuven, Louvain, Belgium; 2 Research Group Work, Organisational and Personnel Psychology (WOPP–O2L), KU Leuven, Louvain, Belgium; 3 Optentia Research Unit, Vaal Campus, North-West University, Vanderbijlpark, South Africa; 4 School of Public Health, University College Cork, Cork, Ireland; 5 National Suicide Research Foundation, University College Cork, Cork, Ireland; 6 Australian Institute for Suicide Research and Prevention, School of Applied Psychology, Griffith University, Brisbane, Australia; 7 Centre Fòrum Research Unit, Hospital del Mar Research Institute, Parc de Salut Mar, Barcelona, Spain; 8 Mental Health Institute Hospital del Mar, Barcelona, Spain; 9 Centro de Investigación Biomédica en Red de Salud Mental (CIBERSAM), Instituto Carlos III, Madrid, Spain; 10 Universitat Pompeu Fab, Barcelona, Spain; 11 Department of Population Health, Faculty of Epidemiology and Population Health, London School of Hygiene and Tropical Medicine, London, UK; 12 National Institute for Health and Welfare (THL), Helsinki, Finland; 13 Institute of Behavioural Sciences, Semmelweis University, Budapest, Hungary; 14 European Alliance against Depression e.V., Leipzig, Germany; 15 Department of Psychiatry, Psychosomatic Medicine and Psychotherapy, University Hospital, Goethe University, Frankfurt Am Main, Germany; 16 University of Stirling, Nursing, Midwifery and Allied Health Professionals Research Unit, Stirling, Scotland, UK; 17 German Depression Foundation, Leipzig, Germany; 18 Depression Research Centre of the German Depression Foundation, Department of Psychiatry, Psychosomatic Medicine and Psychotherapy, University Hospital, Goethe University, Frankfurt am Main, Germany; 19 National Research Centre for the Working Environment, Copenhagen, Denmark; 20 Section of Epidemiology, Department of Public Health, University of Copenhagen, Copenhagen, Denmark; 21 Phrenos Center of Expertise for Severe mental illness, Utrecht, The Netherlands; 22 Altrecht Mental Health Care, Utrecht, The Netherlands; 23 International Association of Suicide Prevention, Washington, DC USA; 24 Academic Center for General Practice, KU Leuven, Louvain, Belgium

**Keywords:** Mental health interventions, Workplace interventions, MENTUPP, Outcome evaluation, Theory of change, SMEs

## Abstract

**Objective:**

Multicomponent interventions are recommendable to achieve the greatest mental health benefits, but are difficult to evaluate due to their complexity. Defining long-term outcomes, arising from a Theory of Change (ToC) and testing them in a pilot phase, is a useful approach to plan a comprehensive and meaningful evaluation later on. This article reports on the pilot results of an outcome evaluation of a complex mental health intervention and examines whether appropriate evaluation measures and indicators have been selected ahead of a clustered randomised control trial (cRCT).

**Methods:**

The MENTUPP pilot is an evidence-based intervention for Small and Medium Enterprises (SMEs) active in three work sectors and nine countries. Based on our ToC, we selected the MENTUPP long-term outcomes, which are reported in this article, are measured with seven validated scales assessing mental wellbeing, burnout, depression, anxiety, stigma towards depression and anxiety, absenteeism and presenteeism. The pilot MENTUPP intervention assessment took place at baseline and at 6 months follow-up.

**Results:**

In total, 25 SMEs were recruited in the MENTUPP pilot and 346 participants completed the validated scales at baseline and 96 at follow-up. Three long-term outcomes significantly improved at follow-up (*p* < 0.05): mental wellbeing, symptoms of anxiety, and personal stigmatising attitudes towards depression and anxiety.

**Conclusions:**

The results of this outcome evaluation suggest that MENTUPP has the potential to strengthen employees’ wellbeing and decrease anxiety symptoms and stigmatising attitudes. Additionally, this study demonstrates the utility of conducting pilot workplace interventions to assess whether appropriate measures and indicators have been selected. Based on the results, the intervention and the evaluation strategy have been optimised.

## Introduction

As described by the World Health Organisation, “mental health is a state of wellbeing in which the individual realizes his or her own abilities, can cope with the normal stresses of life, can work productively and fruitfully, and is able to make a contribution to his or her community” (World Health Organization 2004). Work is considered to be a social determinant of mental health (Wipfli et al. 2021). Positive working conditions are able to protect mental health promoting self-esteem, the sense of being productive and financially safe, and providing the opportunity to people facing psychosocial difficulties to feel that they are included. On the other hand, poor working conditions can raise the opposite effects and have the potential to cause or worsen mental health (Wolrd Health Organization 2022). The relationship between work and mental health can also work vice versa as mental health often leads to absenteeism, presenteeism and eventually to productivity losses (World Health Organization 2022; Cooper and Dewe 2008).

Results from the ^th^ European Working Conditions Survey showed that 6% of the European workers reported very low wellbeing and 15% suffered from anxiety (Eurofound 2017). These people are at increased risk to develop mental health problems (Eurofound 2017; Keyes et al. 2010; Lamers et al. 2015; Santini et al. 2022). The COVID-19 pandemic has further enlarged the prevalence of mental health problems (Hossain et al. 2020; Talevi et al. 2020). Compared to the pre-COVID-19 era, depression or symptoms of depression have increased worldwide with 14% and anxiety or symptoms of anxiety with 13% (Organisqation for Economic Co-operation and Development 2021). Both depression and anxiety, often co-occur with symptoms of burnout; a state mostly related to poor working conditions (Eurofound 2017; Bakker and Demerouti 2007; Maslach et al. 2001). Moreover, people experiencing mental health problems frequently face social exclusion, discrimination and stigmatizing attitudes and behaviors leading to disclosure, which is why addressing these attitudes is often high on the agenda of European public policies (Evans-Lacko et al. 2014).

The fact that mental health and work are strongly intertwined and the current mental health situation of the workforce around the world set the implementation of mental health interventions in the workplace as a priority. Workplace-based mental health interventions are needed not only to improve employees’ mental health but also to reduce the negative economic consequences derived from poor mental health. The workplace itself is an ideal and challenging setting to promote mental health. People spend a lot of time at their workplace where a variety of psychosocial risks can be present in addition to other difficulties including consequences of a national crisis and discrimination based on sociodemographic characteristics (Wolrd Health Organization 2022). SMEs are particularly vulnerable because they are often limited in their capacity to initiate mental health interventions due to lack of resources, time, knowledge and personnel (De Angelis et al. 2020; Beck and Lenhardt 2019), but there is also evidence showing that the lack of complex bureaucratic processes, the feeling of personal accountability, and the potential of teamwork development are elements that can be found in SMEs and are able to facilitate the implementation and efficacy of mental health interventions (McCoy et al. 2014). Despite the interesting contextual characteristics of the SMEs and the fact that they are the backbone of Europe’s economy (Wymenga et al. 2011), the literature on mental health interventions implemented in smaller occupational settings is scarce especially for sectors such as Healthcare that usually consist of larger workforces (Tóth et al. 2023; Greiner et al. 2022; B. Hogg et al. 2021).

A lot of attention has been paid by implementation research to interventions that can promote mental health at work. Previous literature suggests that integrated approaches applied at multiple levels within an organization are recommendable to ensure that the intervention actually leads to better mental wellbeing in the workplace (Cooper and Dewe 2008; Petrie et al. 2018; LaMontagne et al. 2014). Applying multilevel approaches to promote mental health provides the opportunity to intervene across individual and organizational levels within a workplace and use the synergetic effects between them aiming to achieve a better understanding of the effectiveness of the undertaken initiatives (De Angelis et al. 2020). However, there is a lack of evidence on the effectiveness of multilevel interventions, particularly mental health interventions conducted within SMEs (De Angelis et al. 2020; Beck and Lenhardt 2019).

Furthermore, little is known about the effectiveness, usability and transferability of the same multilevel intervention in different work settings and countries (De Angelis et al. 2020; Thornicroft and Patel 2014). Smaller workplaces have fewer hierarchical layers and their workforce structure has more direct connections which is an advantage for policy changes targeting the individual, leader, and organisational level (Linnan 2010). Another difference between work settings is related to employers’ engagement to an intervention. The employers of larger businesses are more used to consider employees’ mental health as one of their responsibilities, whereas employers in smaller workplaces often believe that it is not related or appropriate to their job role and are not convinced that it would be beneficial for their companies (Linnan et al. 2007). Sectoral characteristics should be also taken into consideration as male-dominated organisations have been found to be more hesitant towards mental health interventions (Seaton et al. 2017). Moreover, multilevel interventions are more appropriate when changes are needed at different levels within an organisation. These changes are connected to different aspects of the psychosocial work environment including the effect of the work experience on individuals (micro-level), shared experiences of people working together (meso-level), and the role of environmental features (macro-level) (Martin et al. 2016). However, workplaces do not have the same needs in every level and what works for one may not be a good fit for another. In the same line of thought, the same multilevel intervention may has a different effect amongst implementation countries where the conceptualization of mental health varies and impacts on peoples’ stigmatizing attitudes and help seeking behaviors (Benson and Thistlethwaite 2009). Therefore, it is crucial to provide evidence on the effectiveness of mental health interventions targeting multiple outcomes within occupational settings combining a variety of individual, organizational, sectoral and national characteristics.

MENTUPP (Mental Health Promotion and Intervention in Occupational Settings) is a Horizon 2020 funded project which aims to improve mental health in the workplace by developing a complex evidence-based multilevel intervention. The intervention targets both non-clinical and clinical mental health conditions, and addresses stigmatizing attitudes. The project specifically focuses on SMEs within the construction, health and information and communication technology (ICT) sectors. These sectors have been selected as they have been linked to high levels of stress and negative mental health outcomes (Niedhammer et al. 2021). The intervention has been implemented and evaluated in nine different countries (Albania, Australia, Finland, Germany, Hungary, Ireland, Kosovo, the Netherlands, and Spain), first in a 6-month uncontrolled pilot trial and later on in a large clustered randomized controlled trial (cRCT) (Arensman et al. 2022). Intervention materials and helpful strategies for promoting positive mental health were embedded through the MENTUPP Hub and were provided online to managers, employees and colleagues within participating SMEs (Arensman et al. 2022). The leaders received a targeted intervention with access to material beyond the material for employees. Therefore, leaders are in a dual role in this intervention learning about their own mental health, while they are trying to change the conditions to benefit employees’ mental health.

The aim of the MENTUPP pilot was to test and optimise the intervention, implementation, and evaluation strategy via a comprehensive process evaluation and an outcome evaluation to obtain first results on the effectiveness of the intervention. During the development of the MENTUPP intervention, a program theory was developed based on a participatory approach amongst the researchers involved in the MENTUPP consortium. This resulted in the MENTUPP Theory of Change which visualises the hypothesised causal mechanism of the intervention and enabled us to select the most important long-term, intermediate and proximate outcomes (Tsantila et al. 2023). The MENTUPP intervention is expected to increase the knowledge, skills and attitudes of leaders and employees (proximate outcomes) leading to improved psychosocial working conditions (intermediate outcomes) and eventually resulting in higher mental wellbeing, lower levels of burnout, depression, and anxiety symptoms, less stigmatising attitudes and less absenteeism and presenteeism (long-term outcomes) (Arensman et al. 2022; Tsantila et al. 2023).

The focus of this article is on the outcome evaluation of the long-term outcomes defined by our ToC and assesses whether the desired changes of the intervention were reached. The assessment of the intermediate outcomes and the results of the process evaluation will be reported in a different publication to investigate the causal mechanisms and the circumstances underlying the achieved (or not) change. A more rigorous outcome evaluation, will be conducted via a large-scale currently ongoing cRCT.

This article reports on the long-term outcomes of the pilot trial in relation to four research questions (RQs):

1. Is there an improvement in mental wellbeing, burnout, depression, anxiety, personal stigma towards depression and anxiety, absenteeism and presenteeism after implementing the MENTUPP intervention for six months?
2. Are the changes in the long-term outcomes comparable for the construction, health and ICT sector?
3. Do the changes in the long-term outcomes vary depending on employees’ leadership role in the SME?
4. Did we develop suitable indicators and select appropriate evaluation measures for the assessment of our long-term outcomes?

## Method

### Design

The MENTUPP pilot study followed a mixed methods design collecting quantitative and qualitative data and consisting of a comprehensive process evaluation and an uncontrolled pre–post-outcome evaluation conducted at baseline and at 6-month follow-up.

### Participating SMEs and employees

Nine countries participated in the MENTUPP pilot study, with each country recruiting at least one SME from one specific sector as follows: Construction—Albania, Australia, and Ireland; Health—Hungary, Kosovo, and the Netherlands; ICT—Finland, Germany, and Spain. Enterprises with between 10 and 50 employees were considered as small whereas enterprises occupying 50 to 250 employees were identified as medium-sized (Arensman et al. 2022).

Research Officers (ROs) in each country recruited one or more SMEs for their specified sector. The selection was guided by practical considerations (e.g., approaching an SME with which they already had links, connecting with SME representatives by establishing a connection with workers’ groups, etc.). The aim was to recruit approximately 60–70 employees in the designated sector in each country. Inclusion criteria for the recruitment of employees were persons employed at all levels, part-time and full-time, permanent and non-permanent, inclusion of sub-contractors and agency workers with a contract beyond the follow-up measurement point. No exclusion criteria were defined for employees. Further details with respect to the implementation procedure used during the pilot can be found in Arensman et al. 2022 (Arensman et al. 2022).

### The MENTUPP Hub

The MENTUPP Hub is the online platform (https://www.mentupphub.eu/en/) where the MENTUPP materials including psychoeducational material, animated and real-life videos, audio clips and interactive learning exercises are employed to support mental health and combat stigma within the workplace. The content of the Hub is divided in three thematic areas: (a) components to promote mental wellbeing and prevent stress and burnout, (b) components to prevent and reduce depression and anxiety, and (c) anti-stigma components. The Hub contains not only generic materials for all participants, but also materials tailored to each of the three sectors, and materials to employees and leaders of SMEs. A more detailed description of the intervention components that are provided through the MENTUPP Hub can be found in Arensman et al. (2022) and Tsantila et al. (2023). Important to note is that the original design of the MENTUPP intervention also included face-to-face workshops. However, due to the pandemic, this was not possible anymore and the workshops were replaced by the online interactive learning exercises. For the purpose of the pilot study, the MENTUPP Hub materials were translated in the following languages: Albanian, Dutch, English, German, Hungarian, and Spanish (Finland asked for an English translation).

### Procedure

The ROs in each country followed a standard operating procedure established by the MENTUPP consortium in the recruitment and implementation of the intervention. The ROs were a local steering committee, comprised of key stakeholders of the three sectors, experts in workplace mental health promotion, and academia. An invitation letter to participate in the pilot was sent to SMEs by the ROs, along with an information leaflet detailing basic information about the pilot study and the MENTUPP Hub. An initial meeting with the SME director or a member of the management was set up to discuss the details of participation in the MENTUPP pilot study. ROs assigned to each one of the recruited SMEs established a pilot planning group including at least one employee and one member of the management and then developed an action plan to address psychosocial work environment factors (Arensman et al. 2022).

Introductory sessions were organised with employees and employers approximately two weeks prior to the implementation. During the introductory sessions, the ROs explained the purpose and nature of the study including information on evaluation measures emphasizing that participation is voluntary and that anyone could withdraw at any time. It was noted that employers did not have access to the surveys completed by the employees and that participation would not impact their circumstances of employment in any way, neither positively nor negatively.

Once the participants expressed their interest in the study, they were provided with access to a link leading to the pre-intervention surveys (baseline assessment). First, participants were asked to give informed consent and then they could complete the baseline assessment. To assure participants’ anonymity, a subject-generated identification code (ID-code) was used. The effective use of such an ID-code, allows successful matching of participants across time, depending on the variables that are chosen to generate the ID-code (Yurek et al. 2008). Four questions (variables) were posed to the participants to generate individual ID-codes for the MENTUPP evaluation asking: (1) the first two letters of the official first name of their mother, (2) the day of their birth, (3) the number of biological siblings that they have, and (4) the first two letters of their city or town of birth. Based on the entered digits, an anonymous unique ID-code was generated for every respondent which was used when participants were completing the questionnaires and when they accessed the MENTUPP Hub.

After completing the survey, participants were required to register with the Hub, creating an account to access the materials. SMEs were requested to allow their employees to engage with the Hub during working hours over a six-month intervention period. The time investment to engage with the materials was estimated at eight hours in total which corresponded to an average time use of twenty minutes a week. Following the six-month intervention period, participants were asked to complete the post-intervention surveys (follow-up assessment).

The MENTUPP Hub was opened in March 2021 and remained accessible for six months. Baseline data were collected in March and April 2021 and follow-up data collection was completed in December 2021. Qualtrics https://www.qualtrics.com/nl/core-xm/enquetesoftware/ was used to collect the data of the validated questionnaires and the surveys.

### Ethical considerations

The present study has been approved by each of the local research officer’s institutional ethics committees and is registered with ISRCTN clinical trial registry (ISRCTN14582090) (Arensman et al. 2022).

### Outcome measures

The ToC which we developed to evaluate MENTUPP, identified six proximate outcomes and four intermediate outcomes as hypothesised links leading to the four long-term outcomes of the MENTUPP intervention (Tsantila et al. 2023). This article reports on the evaluation of the long-term outcomes: (1) improved mental wellbeing and reduced burnout, (2) reduced mental illness (in terms of depressive and anxiety symptoms), (3) reduced personal stigma towards mental illness, and (4) reduced productivity losses (in terms of absenteeism and presenteeism) in the SMEs. For each outcome, we formulated a measurable indicator and selected a validated scale to measure it (see Table 1). The long-term outcomes of MENTUPP were assessed using seven selected validated scales.

**Table 1:** Overview of the MENTUPP long-term outcomes indicators

Long-term outcomes	Indicators	Measure
Improved mental wellbeing and reduced burnout of employees and leaders	Increase in wellbeing at follow-up	WHO-5 OLBI
Reduced mental illness in employees and leaders	Reduced depressive and anxiety symptoms (including suicidality) at follow-up	PHQ-ADS consisting of PHQ-9 & GAD-7
Reduced personal stigma towards mental illness in the workplace	Reduced personal stigma at follow-up	DSS
Reduced productivity losses in the SMEs	Reduced absenteeism and presenteeism	SPS-6 WPAI-GH

In our outcome evaluation, we also included some socio-demographic and work-related characteristics of the sample including age, gender, educational level, nationality, type of contract, employment rate and leadership role. The latter variable, “leadership role”, was based on respondents’ responses to a question scaled on a 11-point Likert scale to what extent they had a leading role in their work task (0 = no leading role at all; 10 = full time leading role). Scores ranging from 0 to 3 were labelled as “low leadership role”, scores ranging from 4 to 6 as “medium leadership role”, and scores ranging from 6 to 10 as “large leadership role”.

The Oldenburg Burnout Inventory (OLBI) was developed by Demerouti and colleagues (Demerouti et al. 2001) and measures burnout. The questionnaire consists of two subscales which are rated on a four-point Likert scale. The exhaustion subscale consists of eight items and measures general feelings of emptiness, overtaxing from work, a strong need for rest, and a state of physical exhaustion. The disengagement subscale also consists of eight items and measures distancing oneself from the object and the content of one’s work and adopting negative, cynical attitudes and behaviours towards one’s work in general. Both subscales consist of four positively worded items and four negatively worded items. The negatively worded items are reversed during scoring. For each subscale, a mean value across all items is computed, with resulting scale scores ranging from 1 to 4 (i.e. 1 = not exhausted/not disengaged and 4 = completely exhausted/completely disengaged). Its structure is essentially invariant across occupational groups and it demonstrates acceptable reliability and validity (J. Halbesleben and Demerouti 2005a).

For the outcome “reduced mental illness”, we measured symptoms of depression and anxiety using the Patient Health Questionnaire Anxiety and Depression Scale (PHQ-ADS). The PHQ-ADS is developed by Kroenke and colleagues (Kroenke et al. 2016) and is a composite measure of depression and anxiety which demonstrated high reliability and strong validity. It consists of the nine items of the Patient Health Questionnaire depression scale (PHQ-9) and the seven items of the Generalized Anxiety Disorder scale (GAD-7) rated on a four-point Likert scale. Respondents are asked to indicate on a four-point Likert scale (0: not at all; 1: several days; 2: more than half the days; 3: nearly every day) how often each symptom bothered them over the past 2 weeks. The PHQ-9 score is calculated by adding together the nine item scores and ranges from 0 to 27, with higher scores representing more severe symptoms of depression. The total score on the GAD-7 is obtained by adding together the seven item scores and ranges from 0 to 21, with higher scores representing more severe anxiety.

Stigmatizing attitudes towards depression and anxiety were measured with the Personal Stigma subscale of the Depression Stigma Scale (DSS) for which we slightly rephrased the items to assess stigma towards both depression and anxiety. The DSS is a valid and reliable instrument developed by Griffiths and colleagues (K. M. Griffiths et al. 2004) and originally measures stigma towards depression. The Personal Stigma subscale consists of nine items and measures respondents’ personal attitudes towards depression and anxiety. Participants respond on each of the nine items via a five-point Likert scale ranging from “strongly disagree” (score 1) to “strongly agree” (score 5). Subscale scores are then calculated by summing the nine item scores, which results in a score ranging from 9 to 45, with higher scores indicating more stigmatizing attitudes.

Finally, the impact of the intervention on productivity losses was assessed by measuring absenteeism and presenteeism. To measure absenteeism (the percentage of work time missed because of one’s health in the past 7 days), we selected two items of the Work Productivity and Activity Impairment—General Health V2.0 (WPAI-GH 2.0). Respondents are asked to indicate the number of hours they missed from work because of their health problems and the number of hours they actually worked the past 7 days. The WPAI-GH 2.0 was developed by Reilly associates and is a validated instrument commonly used to measure work productivity losses due to mental illness (Asami and Okumura 2015; Erickson et al. 2009). The percentage of work time lost due to health-related problems, is calculated by dividing the hours missed by the sum of the hours missed and the hours actually worked. This ratio is accordingly multiplied by 100. WPAI outcomes are expressed as impairment percentages, with a higher percentage indicating more absenteeism.

For presenteeism, the Stanford Presenteeism Scale (SPS-6) was used. The SPS-6 was developed by Koopman and colleagues (C. Koopman et al. 2002a) and measures employees’ perceived ability to concentrate on work tasks despite the distractions of health difficulties. Respondents are asked to describe their work experiences in the past month by means of six items via a five-point Likert scale ranging from “strongly disagree” (score 1) to “strongly agree” (score 5). The SPS-6 score is calculated by adding all six items and ranges from 6 to 30. Scores between 6 and 18 represent more presenteeism (i.e., reduced ability to work productively and reduced work performance) and scores between 19 and 30 denote better performance at work. The SPS-6 demonstrates a high level of validity and reliability to measure health-related productivity in diverse employee populations (Turpin et al. 2004).

An overview of the psychometric properties of the validated scales can be found in Table 2. For the purpose of the pilot study, the validated instruments were translated in the eight MENTUPP languages: Albanian, Kosovan Albanian, Dutch, English, Finnish, German, Hungarian, and Spanish. When available, validated translations of the questionnaires, were used. For the scales where a validated translation was not available, the ROs of the MENTUPP countries relied on a back translation procedure to translate the items (Brislin 1970). Table 2 provides an overview of the available validated translations.

**Table 2:** Overview of psychometric properties and available translations of the seven validated scales

Validated scale	Short-name	Internal consistency	Test–retest reliability	Validated translation
World Health Organization–Five Wellbeing Index	WHO-5	*Cronbach α* = 0.85 (Omani-Samani et al. 2019)	Pearson’s correlation = 0.81 (Schougaard et al. 2018)	Available for all languages
Oldenburg burnout inventory	OLBI	*Cronbach α* > 0.70 (J.R.B. Halbesleben and Demerouti 2005b)	Exhaustion subscale: Pearson’s Correlation = 0.51 Disengagement subscale: Pearson’s correlation = 0.34 (J.R.B. Halbesleben and Demerouti 2005b)	Available for all languages except for Albanian
Patient health questionnaire–anxiety and depression scale	PHQ-ADS	*Cronbach α* > 0.80 (Kroenke et al. 2016)	PHQ-9: Pearson’s correlation = 0.94 (Zuithoff et al. 2010) GAD-7: Pearson’s correlation = 0.83 (Spitzer et al. 2006)	Available for all languages except for Albanian
Depression Stigma Scale	DSS	Personal stigma subscale: *Cronbach α* = 0.77 (Kathleen M. Griffiths and Jorm 2008)	Personal stigma subscale: Pearson’s correlation > 0.66 (K. M. Griffiths et al. 2004)	Available for all languages except for Albanian
Work productivity and activity impairment – general health	WPAI-GH	*Cronbach α* = 0.74 (Ciconelli et al. 2006)	Pearson’s correlation > 0.69 (Lofland and Frick 2004)	Available for all languages except for Albanian
Stanford presenteeism scale	SPS-6	*Cronbach α* = 0.80 (Cheryl Koopman et al. 2002b)	Spearman’s correlation = 0.82 (Hutting et al. 2014)	Available for all languages except for Albanian, Hungarian and German

## Data analysis

In the current study, the amount of missing data was high (72.3%). Jakobsen et al. (2017) provide a practical guide to handle missing data when longitudinal data with a large proportion of dropout (more than 40% of people dropped out of a study).are being analysed. In line with their recommendations, we relied on a complete case analysis to analyse the data, we transparently described the extent and the nature of the dropout in the results section, and we highlighted the limitations of our results in the discussion section. In complete case analyses, only participants with a complete set of outcome data are included in the statistical analyses (Jakobsen et al. 2017; Clark and Altman 2003).

Statistical analyses were carried out using SPSS 28.0. First, SMEs’ and respondents’ characteristics were examined relying on descriptive statistics. Second, a dropout analysis was conducted comparing the dropout group to the group of respondents participating at baseline and follow-up on a range of variables by using independent sample t-tests and chi-square tests. Cohen’s d or phi coefficients were calculated as an indicator of effect sizes.

Third, to examine whether the intervention had an impact on the long-term outcomes, a repeated measures ANOVA was conducted on the seven scale scores with Time (baseline vs. follow-up) being entered as a within-subjects factor and with Sector (construction vs. health vs. ICT), Leadership Role (low vs. medium vs. large leadership role) and Country (Albania vs. Finland vs. Germany vs. Hungary vs. Kosovo vs. the Netherlands vs. Spain) being entered as three between-subjects factors.

## Results

### Characteristics of participating SMEs

Across the nine intervention countries, a total of 25 SMEs was recruited to participate in the pilot trial. Table 3 presents the distribution of the SMEs by size, country and sector. In countries with a lower number of participating SMEs, there was a relatively high uptake rate of MENTUPP in the first SME that was recruited, making further recruitment for the pilot study redundant. Eleven SMEs were active in the health sector, whereas the construction and ICT sectors each counted seven participating SMEs. One quarter of the participating SMEs were family businesses.

**Table 3:** Number of participating SMEs per size, country, and sector

Country	Sector	*N* and size of SMEs
Albania	Construction	3 Small sized
Australia	Construction	1 Small and 1 Medium sized
Finland	ICT	1 Medium sized
Germany	ICT	1 Medium sized
Hungary	Health	4 Small & 1Medium sized
Ireland	Construction	2 Medium sized
Kosovo	Health	5 Small sized
Netherlands	Health	1 Medium sized
Spain	ICT	3 Small & 2 Medium sized

### Characteristics of participating employees and drop-out group

In total, 346 respondents completed the seven validated scales at baseline, but only 96 participants at follow-up. Table 4 displays the participants’ characteristics, their baseline mean scores on the outcome scales and summarises the number of respondents per sector, leadership role and country for all respondents (baseline), for respondents who completed both baseline and follow-up surveys (complete cases) and for respondents who dropped out at follow-up. The mean age of the respondents who participated at baseline and at follow-up was 38 years old. Our sample was evenly distributed between genders and 88.7% had a higher educational level. The majority of the participants (46.9%) worked in the ICT sector, 31.3% in the Health sector and 21.9% in Construction. Most of the respondents were from Finland (36.5%), Albania (21.9%), and Kosovo (20.8%). Importantly, there were no complete cases for Australia and Ireland.

**Table 4:** Participants’ characteristics, baseline mean scores on the outcome scales, and number of respondents that completed the seven validated scales at baseline, at baseline and at follow-up, and that dropped out

		Participated at baseline *N* = 364 Mean (SD)/%	Drop-out group *N* = 268 Mean (SD)/%	Complete cases *N* = 96 Mean (SD)/%
Age		38.3 (11.2)	38.4 (11.4)	38 (10.4)
Gender	Male	63.5%	67.9%	51%
	Female	36.5%	32.1%	49%
Country	Albania	17.3%	15.7%	21.9%
	Australia	17%	23.1%	0%
	Finland	25%	20.9%	36.5%
	Germany	3.8%	4.1%	3.1%
	Hungary	8.5%	9.7%	5.2%
	Ireland	4.7%	6.3%	0%
	Kosovo	9.1%	4.9%	20.8%
	Netherlands	4.1%	3%	7.3%
	Spain	10.4%	12.3%	5.2%
Sector	Construction	38.7%	44.8%	21.9%
	Health	20.9%	17.2%	31.3%
	ICT	40.4%	38.1%	46.9%
Education level	Primary education	3.6%	3.7%	3.1%
	Lower secondary education	14.8%	17.2%	8.3%
	Upper secondary or post-secondary education	22.8%	24.3%	18.9%
	Tertiary education	58.8%	54.9%	69.8%
Scores in long-term outcomes scales	Wellbeing	58.8 (18.6)	58 (19)	61 (17.2)
	Burnout	37 (6.4)	37.4 (6.6)	36 (5.8)
	Depression	6.3 (4.7)	6.6 (5)	5.3 (3.8)
	Anxiety	5.6 (4.5)	5.8 (4.5)	5.2 (4.4)
	Personal stigma	21.2 (7.6)	20.9 (7.6)	22.2 (7.6)
	Absenteeism	4 (12.6)	4.2 (11.9)	3.4 (14.6)
	Presenteeism	21.5 (4.7)	21.3 (4.7)	22 (4.4)

No significant differences were found comparing the age and the educational level of the complete cases and those who dropped out. However, significant differences were found regarding gender [*x*^*2*^ (1) = 0.867, *p* < 0.05], country [*x*^*2*^ (8) = 65, *p* < 0.05], and sector [*x*^*2*^ (8) = 65, *p* < 0.05] between the two groups. The majority of the people who dropped out were men (67.9%), whereas the percentage of men in the complete cases was 51%. A high number of participants who dropped out were from Australia (23.1%), Finland (20.9%), and Albania (15.7%). While there were no complete cases from Australia, the percentages of complete cases in Finland (36.5%), and Albania (21.9%) were higher than those in the dropout group. Finally, the vast majority of the respondents who dropped out at follow-up worked in Construction (44.8%), when people working in ICT had the highest participation percentage (46.9%) at follow-up. Equivalence could not be established for all variables which were included in the dropout analysis. The effect sizes regarding the differences between the two groups were within the range of what is considered small for gender and sector (*Phi* = 0.154 and *Phi* = 0.220), and moderate for country (*Phi* = 0.423).

No significant differences were found in the baseline mean scores on the outcome scales between the respondents who dropped out and those who did not concerning wellbeing, burnout, anxiety, personal stigma, absenteeism and presenteeism. However, a significant difference was found between the two groups regarding depression [*F* (1362) = 5.8, *p* < 0.05] showing that people who dropped out indicated more symptoms of depression [*EMs* 6.6 (*SE* = 4.9)] than those who did not drop out [*EMs* 5.3 (*SE* = 3.7)] at baseline.

### Impact of MENTUPP on long-term outcomes

#### Mental wellbeing scale (WHO-5)

The main effect of time reached significance, with* F*(1,74) = 5.35, *p* < 0.05, and *η*^*2*^ = 0.067 indicating a medium effect size. Further exploration showed that estimated means (*EMs*) increased from 57 at baseline (standard error (*SE*) = 2.4) to 63 at follow-up (*SE* = 2.7), suggesting that wellbeing had been improved at follow-up. Results showed a significant main effect of country *F*(5,74) = 2.63, *p* < 0.05, with Kosovo and Albania scoring higher on the WHO-5 [*EMs* 71.2 (*SE* = 5) and 69.2 (*SE* = 4), respectively] and Spain and the Netherlands scoring lower [*EMs* 54.2 (*SE* = 7) and 49.6 (*SE* = 6), respectively]. Neither the two-way interaction between time and sector [*F* < 1] nor the two-way interaction between time and leadership role [*F*(2,74) = 1.8, *p* = 0.17] reached significance.

#### Burnout scale (OLBI)

The main effect of time reached neither significance for the exhaustion subscale, with *F*(1,74) = 0.002, *p* = 0.96, nor for the disengagement subscale, with *F*(1,74) = 1.58, *p* = 0.21. For none of the two subscales, the two-way interactions between time and country [with *F* < 1 for both subscales], time and sector [with *F* < 1 for the exhaustion subscale and *F*(1,74) = 1.52, *p* = 0.22 for the disengagement subscale], and time and leadership role [with *F* < 1 for both subscales] was significant.

#### Depression scale (PHQ-9)

The main effect of time was not significant, with *F*(1,74) = 2.63, *p* = 0.19 [*EM* of 5.6 (*SE* = 0.6) at baseline and *EM* of 4.7 (*SE* = 0.7) at follow-up]. Also, the two-way interactions between time and country [*F* < 1], time and sector [*F*(1,74) = 1.87, *p* = 0.17], and time and leadership role [*F* < 1] did not reach statistical significance.

#### Anxiety scale (GAD-7)

The main effect of time reached significance, with *F*(1,74) = 10.1, *p* < 0.05, and *η*^*2*^ = 0.12 indicating a large size effect. Further exploration showed that *EMs* decreased from of 5.8 (*SE* = 0.6) at baseline to 3.7 (*SE* = 0.7) showing that the level of anxiety has been improved at follow-up. However, significance was not observed for any of the two-way interactions, suggesting that the decrease in anxiety did not differ between countries [*F*(5,74) = 1.41, *p* = 0.22], sectors [*F* < 1], and leadership roles [*F* < 1].

#### Personal stigma scale (DSS)

The main effect of time reached significance for the personalised stigma scale, with *F*(1,74) = 5.46, *p* < 0.05, and *η*^*2*^ = 0.069 indicating a medium size effect. Further exploration showed more favourable attitudes towards depression and anxiety at follow-up (*EM* = 20.6 and *SE* = 0.9) than at baseline (*EM* = 22.5 and *SE* = 1). Significance was not observed for the two-way interactions time and country [*F* < 1], time and sector [*F* < 1], and time and leadership role [*F* < 1].

#### Absenteeism scale (WPAI-GH)

The main effect of time was not significant, with *F*(1,71) = 0.1, *p* = 0.75 (*EM* = 3.4, *SE* = 2.5 at baseline and *EM* of 2.5, *SE* = 2 at follow-up). In addition, the two-way interactions between time on the one hand and country, sector and leadership role on the other hand did not reach significance [with *F* < 1 for all three two-way interactions].

#### Presenteeism scale (SPS-6)

The main effect of time did not reach statistical significance, with *F* < 1 [*EM* = 22 (*SE* = 0.7) at baseline and *EM* = 22.3 (*SE* = 0.7) at follow-up]. In addition, none of the two-way interactions between time on one hand and country, sector and leadership role on the other hand reached significance [with *F* < 1 for all three two-way interactions] (Table 5).

**Table 5:** Estimated means, standard errors, and confidence intervals of the complete cases on the seven scales at baseline and at follow-up

		Baseline (*N *= 96)				Follow-up (*N* = 96)			
				95% CI				95% CI	
Measured construct	Scales’ range	EM	SE	LL	UL	EM	SE	LL	UL
Mental wellbeing*	0–100	57	2.4	52.1	61.7	63	2.7	57.8	68.7
Burnout	16–64	36	0.85	34.2	37.6	36	0.75	34.6	37.6
Depression	0–27	5.6	0.6	4.5	6.8	4.7	0.7	3.2	6.3
Anxiety*	0–21	5.8	0.6	4.6	7	3.7	0.7	2.4	5
Personal stigma*	9–45	22.5	1	20.5	24.5	20.6	0.9	18.8	22.5
Absenteeism	No specified range	3.4	2.5	− 1.6	8.3	2.5	2	− 1.6	6.6
Presenteeism	6–30	22	0.7	20.6	23.2	22.3	0.7	21	23.7

*a significant effect of time was found (*p* < .05)

Note: *EM* estimated mean; *SD* standard error; *CI* confidence interval; *LL* lower limit; *UL* upper limit

### Strengths and limitations

A particular strength of this study is that it evaluates a workplace intervention that was implemented internationally and in three different sectors including people from various job roles and focusing on the previously neglected evidence of workplace mental health interventions in SMEs. The MENTUPP pilot helped us to examine impact differences between the different contexts and report on the applicability of our intervention among them. This is not only useful to inform the upcoming MENTUPP cRCT, but also for future research on global complex mental health interventions which is an underexplored field (Thornicroft and Patel 2014).

A second strength of our study is that we relied on a theory driven approach to evaluate our complex intervention. In a first phase, we developed a ToC which visualizes the rationale and the mechanism of change of our intervention and describes on which outcomes MENTUPP is expected to generate an effect (Tsantila et al. 2023). This approach allowed us to make a well-considered selection of outcomes that forms the heart of our outcome evaluation strategy. Next, we linked every outcome to specific indicators and selected appropriate scales to measure them. The results of this pilot study showed that we were able to observe changes for several of our outcomes, providing initial evidence that we selected meaningful outcomes and indicators and that we used for many of these indicators appropriate measures to capture them.

Another advantage of this outcome evaluation study, is that it helped us to further optimize the content of the MENTUPP intervention, the implementation process and the evaluation strategy. We obtained initial evidence that mental wellbeing, anxiety and personal stigma towards depression and anxiety can be changed through our intervention and that several evaluation measures that we selected are able to capture this change. We believe that our intervention has potential to have an even greater impact on mental health and productivity loss if leaders receive during implementation more practical guidance on how to conduct positive changes in workload, emotional demands and conflicts in the workplace. Due to the COVID-19 pandemic, the amount of guidance provided during implementation was limited. Ahead of the MENTUPP cRCT, we optimized the materials embedded in the MENTUPP Hub, we worked out a more intense and interactive implementation approach focusing on important psychosocial factors and we selected more appropriate evaluation measures to assess burnout and productivity losses.

A major limitation of this study, is the high dropout rate of respondents completing the follow-up measures. The low response rate does not allow a confirmatory interpretation of our findings. While some of our results are encouraging, we consider them as preliminary and are aware that more advanced research is needed to which the cRCT will contribute. The high dropout in this pilot study may have different reasons. First, it is possible that the evaluation part was too extensive for employees to complete. For the cRCT, the number of items used for the evaluation is halved. Second, COVID-19 has genuinely disrupted the implementation process of the MENTUPP pilot study and we are hopeful that the cRCT is safe from any COVID-19 measures. Third, it is also possible that the intervention did not match with the needs of leaders. Fourth, according to our findings it is more possible for males, and people working in the Construction to drop out. Moreover, through the dropout analysis we conducted, we noticed that almost 70% of the complete cases had a tertiary education. However, it is important to mention that this is perhaps not representative of the participating countries as according to evidence the percentage of people with tertiary education varies across them (OECD 2016; UNESCO 2022). A closer look on how the level of education is distributed across countries and sectors of the complete cases can be found in Annex 1. Within the context of the pilot study, a comprehensive process evaluation was conducted which will provide more details on the strengths and difficulties of the implementation and the dropout rate (Arensman et al. 2022).

Finally, the validated scales we selected to assess the long-term outcomes of the intervention in their majority consist of negatively worded items while research focuses on the importance of the inclusion of positive aspects when mental health is evaluated (Bieda et al. 2017). An exception is the WHO-5 (wellbeing) scale which uses only positively worded items and the OLBI (burnout) which uses a mix of positively and negatively worded items. However, the use of mixed scales including positive and negative items or the total exclusion of negatively worded items is also debatable. Especially, when the attributes assessed are negative in nature (e.g. depression) (Chyung and Shamsy 2018).

## Discussion

Regarding our first research question, a positive change in mental wellbeing, symptoms of anxiety and personal stigma towards depression and anxiety was found. These findings support frameworks that postulated that workplace interventions directed at both the individual and organisational level have a positive impact on mental wellbeing (Martin et al. 2019; LaMontagne et al. 2014; Petrie et al. 2018). Moreover, research has shown that multi-component workplace interventions utilizing several techniques tend to be more effective towards common mental health disorders such as anxiety (Joyce et al. 2016). The results are also consistent with other findings demonstrating that mental health interventions in the workplace are able to induce small improvements in anxiety and depression (Martin and Cocker 2009). Tentative evidence is also available reporting that anti-stigma components should be integrated in workplace interventions as they can have a positive impact on employees’ knowledge, attitudes and behaviour towards mental illness (Hanisch et al. 2016; Bridget Hogg et al. 2022) and eventually on mental health itself (Kitchener and Jorm 2004; Gould et al. 2007). The obtained results showing that MENTUPP has the potential to reduce personal stigma are congruent with those found by previous research about the effect of workplace mental health interventions on stigmatizing attitudes towards depression and anxiety (Kathleen M. Griffiths et al. 2016). Furthermore, our study adds to existing knowledge by incorporating an anti-stigma component as a part of a wider program with significant positive effects on personal stigma (Szeto and Dobson 2010). Hence, we found promising results demonstrating that MENTUPP has potential to produce positive changes in several of our long-term outcomes. We obtained and invested even more on the anti-stigma materials which include multiple intervention techniques such as psychoeducation, interactive skills training exercises, and peer support activities. These materials are able to contribute to structural changes in the SMEs such as the promotion of communication strategies for supporting employees, the creation of a more inclusive working environment leading to lower levels of personal (self-stigma) and perceived (social) stigma and, respectively, to more positive mental health outcomes (Tóth et al. 2023). Importantly, our intervention which was delivered totally online shows the ability to achieve mental health outcomes in the working sectors of SMEs. These populations cannot be easily reached by mental health interventions. We also conclude that we have selected appropriate output indicators and evaluation measures to capture changes in mental wellbeing, symptoms of anxiety, stigmatising attitudes towards depression and anxiety.

Nevertheless, no significant effects were observed for burnout, symptoms of depression and productivity losses in terms of absenteeism and presenteeism. The absence of significant effects here may have various reasons. In general terms, the implementation context of the pilot study was not ideal, as it was conducted during the height of the COVID-19 pandemic, which impacted considerably on the implementation process. Communications with the SMEs occurred mostly online and many employees worked from home during that period. More specifically for burnout, it is possible that the 6-month implementation period was too short to induce improvements during the pandemic (Ghahramani et al. 2021). Research with multilevel interventions like MENTUPP (integrating person- and organization-oriented approaches) has shown that effects on burnout are stronger when the intervention lasts longer (Awa and Walter 2010). Implementing structural workplace changes such as adapting the level of job demands or increasing employee control requires a certain amount of time, and thus changes in terms of burnout are more visible when a more extensive follow-up period is used. In addition, the COVID-related restrictions and the associated increased work demands may have led to increased burnout symptoms, especially, in the healthcare sector, hereby counteracting any potential positive effects of the intervention.

Based on the pilot results, we have no reason to question the inclusion of any of the selected outcomes specified in our ToC. For some of the outcomes, we do have doubts on whether we selected the most appropriate indicator for our study especially for the outcomes ‘burnout’ and ‘productivity losses’. Ahead of the large-scale study, we propose a modified operationalization for both. The OLBI questionnaire, which we used to measure burnout, has been criticized by researchers in the past as it assesses disengagement and exhaustion using not only negatively but also positively worded items. Positively worded questions may be more suited to capture work-engagement rather than burnout (Schaufeli and De Witte 2020). Moreover, although OLBI has been used in a lot of studies its test–retest reliability is considered fair (Matheson 2019). Therefore, it is possible that the OLBI was not sensitive enough to pick up changes in burnout induced by our intervention. For burnout, we propose to use the Burnout Assessment Tool (BAT) instead of the OLBI as a measure. The BAT has been shown to have a higher internal consistency (*Cronbach α* > 0.90), and excellent test–retest reliability (Pearson’s Correlation = 0.60 to 0.75), and relies on an updated conceptualization of burnout incorporating four core dimensions: (1) exhaustion, (2) emotional impairment, (3) cognitive impairment, and 4) mental distance using only negatively worded items. The scale is able to screen employees who are at risk of burnout and diagnose those who already have burnout. (Schaufeli and De Witte 2020).

Productivity losses, as measured with the SPS-6 and the WPAI-GH, showed neither improvement of absenteeism nor improvement of presenteeism at follow-up. As it is presented in Table 2, the WPAI-GH demonstrates a good but not an excellent score on test–retest reliability which may have impacted on our findings. However, this could not be the case for the SPS-6 which has excellent psychometric properties (see Table 2). The short follow-up of the pilot study could be responsible for the absence of any effect on these outcomes. Another reasoning could be that presenteeism and absenteeism are strongly related to psychosocial work conditions such as high job demands, high emotional demands, low job autonomy, low job control, low opportunities for development, and low social support in the workplace (van den Heuvel et al. 2010; Kivimäki et al. 1997; Harter Griep et al. 2010; Janssens et al. 2016). The MENTUPP pilot educates leaders to identify detrimental working conditions and develop a plan to change them. The emphasis on improving communication and social support is considered a strong asset of the MENTUPP intervention. However, more practical information is possibly needed to encourage companies to ameliorate aspects such as workload, emotional demands and conflicts in the workplace, it could be possible to improve symptoms of burnout (Nuebling et al. 2013; Bria and Dumitrascu 2012) and reduce absenteeism and presenteeism. Also, productivity loss due to mental ill-health is a difficult construct to measure and it is possible that the SPS-6 and the WPAI-GH are not appropriate measures for these constructs (Mattke et al. 2007; Lensberg et al. 2013). For productivity losses which translate into the indicators ‘absenteeism’ and ‘presenteeism’, we suggest using customized items that are more aligned with what we want to know. For the cRCT, a more elaborate approach will be used to conduct the economic evaluation of our intervention including three different perspectives: an employer perspective, a healthcare perspective, and a societal perspective. The employer perspective considers the costs of mental health issues that are borne by the employer in terms of productivity loss as well as the costs of implementing the intervention that are paid by the employer and the potential healthcare costs borne by the employer. The healthcare perspective only includes costs that are expended on healthcare services funded by the health system. The societal perspective includes all costs borne by the whole of society, including productivity costs or other costs not borne by the health system or the employer (Gaillard et al. 2020).

For depression, it is of importance that the scores of participants on the PHQ-9 were already very low at baseline leaving little room for improvement. The PHQ-9 is a valid scale to assess the efficacy of interventions targeting depression (Oehler et al. 2021, 2020) with excellent psychometric properties (see Table 2). Therefore, we have no reason to doubt the scale’s sensitivity to detect changes in depression.

With respect to the second and third research questions concerning differences in the long-term outcomes between the involved work sectors and leadership roles, we did not find significant differences in the long-term outcomes between them. We consider this to be a positive result as we developed a tailored intervention that would be able to address the different needs of people coming from three work sectors and having low or high leadership roles within the organisations.

## Conclusion

The study outcomes show that mental health interventions such as the one developed by MENTUPP have the potential to improve mental wellbeing, anxiety symptoms and stigma towards depression and anxiety. Importantly, this study contributes to the limited empirical evidence available for SMEs which is an underexplored field in literature (Hogg et al. 2021, 2022). Moreover, we argue that when targeting more structural changes in the workplace through mental health interventions, we increase the possibility to achieve positive outcomes in burnout symptoms and productivity losses. Thus, we believe that the optimization of the intervention should follow this line of thought, whereas we have no reason to focus on further tailoring per sector and job role. The intervention components have been enriched in order to promote the identification and management of high demands in the workplace, the resolution of conflicts, the increasement of influence and control and the design of plans to promote job redesign. In addition, a preliminary discussion with people working in an organisation where an intervention is planned to be implemented can be proved very helpful to identify the mental health needs of the working population and support us when developing it.

Τhe outcomes targeted by MENTUPP remain the same for the cRCT and we defined valid and feasible assessment for our complex intervention. Our ToC itself has been optimized and will provide guidance not only to the MENTUPP trial but also to the development, implementation and evaluation of future projects of high complexity. This way, our study adds to the evidence required to conduct high-quality evaluations (Paterson et al. 2021). More research should be conducted using pilot studies of integrated mental health interventions in the workplace as this will lead to a better perception of the mechanisms of change underlying them and, respectively, to more successful trials.

## Data Availability

The datasets generated and/or analysed during the current study are available from the corresponding author on reasonable request. When the project will be completed, all of our data will be saved together in an open access repository. The journal will be informed about the name of the repository as soon as it will be available.

## Annex 1 Education level of complete cases per country and sector

**Table:**

	*N*/% Primary education		*N*/% Lower secondary education		*N*/% Upper secondary or post-secondary education		*N*/% Tertiary education	
Country								
Albania	2	9.5	6	28.6	5	23.8	8	38.1
Finland	1	2.9	0	0	1	2.9	33	94.3
Germany	0	0	1	33.3	0	0	2	66.7
Hungary	0	0	0	0	0	0	5	10
Kosovo	0	0	0	0	12	60	8	40
Netherlands	0	0	0	0	0	0	7	100
Spain	0	0	1	20	0	0	4	80
Sector								
Construction	2	9.5	6	28.6	5	23.8	8	38.1
Health	0	0	0	0	12	40	18	60
ICT	1	2.2	2	4.4	1	2.2	41	91.1
