# Correction: Outcome assessment of a complex mental health intervention in the workplace. Results from the MENTUPP pilot study

**DOI:** 10.1007/s00420-023-02016-0

**Published:** 2023-10-11

**Authors:** Fotini Tsantila, Evelien Coppens, Hans De Witte, Ella Arensman, Benedikt Amann, Arlinda Cerga-Pashoja, Paul Corcoran, Johanna Creswell-Smith, Grace Cully, Monika Ditta Toth, Birgit Greiner, Eve Griffin, Ulrich Hegerl, Carolyn Holland, Caleb Leduc, Mallorie Leduc, Doireann Ni Dhalaigh, Cliodhna O’Brien, Charlotte Paterson, György Purebl, Hanna Reich, Victoria Ross, Reiner Rugulies, Sarita Sanches, Katherine Thompson, Chantal Van Audenhove, MENTUPP consortium members, Kahar Abula, Birgit Aust, Laura Cox, Luigia D’Alessandro, Grace Davey, Lars De Winter, Kim Dooyoung, Asmae Doukani, Arilda Dushaj, Naim Fanaj, Stefan Hackel, Bridget Hogg, Sharna Mathieu, Margaret Maxwell, Ana Moreno- Alcazar, Karen Mulcahy, Doireann Ni Dhalaigh, Ainslie O’ Connor, Wendy Orchard, Gentiana Qirjako, Saara Rapeli, Sarita Sanches, Andras Szekely, Jaap Van Weeghel, Kristian Wahlbeck, Eva Zsak

**Affiliations:** 1 Centre for Care Research and Consultancy, LUCAS, KU Leuven, Louvain, Belgium; 2 Research Group Work, Organisational and Personnel Psychology (WOPP–O2L), KU Leuven, Louvain, Belgium; 3 Optentia Research Unit, Vaal Campus, North-West University, Vanderbijlpark, South Africa; 4 School of Public Health, University College Cork, Cork, Ireland; 5 National Suicide Research Foundation, University College Cork, Cork, Ireland; 6 Australian Institute for Suicide Research and Prevention, School of Applied Psychology, Griffith University, Brisbane, Australia; 7 Centre Fòrum Research Unit, Hospital del Mar Research Institute, Parc de Salut Mar, Barcelona, Spain; 8 Mental Health Institute Hospital del Mar, Barcelona, Spain; 9 Centro de Investigación Biomédica en Red de Salud Mental (CIBERSAM), Instituto Carlos III, Madrid, Spain; 10 Universitat Pompeu Fab, Barcelona, Spain; 11 Department of Population Health, Faculty of Epidemiology and Population Health, London School of Hygiene and Tropical Medicine, London, UK; 12 National Institute for Health and Welfare (THL), Helsinki, Finland; 13 Institute of Behavioural Sciences, Semmelweis University, Budapest, Hungary; 14 European Alliance against Depression e.V., Leipzig, Germany; 15 Department of Psychiatry, Psychosomatic Medicine and Psychotherapy, University Hospital, Goethe University, Frankfurt Am Main, Germany; 16 University of Stirling, Nursing, Midwifery and Allied Health Professionals Research Unit, Stirling, Scotland, UK; 17 German Depression Foundation, Leipzig, Germany; 18 Depression Research Centre of the German Depression Foundation, Department of Psychiatry, Psychosomatic Medicine and Psychotherapy, University Hospital, Goethe University, Frankfurt am Main, Germany; 19 National Research Centre for the Working Environment, Copenhagen, Denmark; 20 Section of Epidemiology, Department of Public Health, University of Copenhagen, Copenhagen, Denmark; 21 Phrenos Center of Expertise for Severe mental illness, Utrecht, The Netherlands; 22 Altrecht Mental Health Care, Utrecht, The Netherlands; 23 International Association of Suicide Prevention, Washington, DC USA; 24 Academic Center for General Practice, KU Leuven, Louvain, Belgium

**Correction: International Archives of Occupational and Environmental Health (2023) 96:1149–1165** 10.1007/s00420-023-01996-3

In the original version of this article, the given and family names of all authors in the author group were interchanged. The correct names are given below.

Fotini Tsantila · Evelien Coppens · Hans De Witte · Ella Arensman · Benedikt Amann · Arlinda Cerga-Pashoja · Paul Corcoran · Johanna Creswell-Smith · Grace Cully · Monika Ditta Toth · Birgit Greiner · Eve Grifn · Ulrich Hegerl · Carolyn Holland · Caleb Leduc · Mallorie Leduc · Doireann Ni Dhalaigh · Cliodhna O’Brien · Charlotte Paterson · György Purebl · Hanna Reich · Victoria Ross · Reiner Rugulies · Sarita Sanches · Katherine Thompson · Chantal Van Audenhove · MENTUPP consortium members

The original article has been corrected.

